# Sarcoidosis mimicking nodal manifestations of marginal zone lymphoma

**DOI:** 10.1007/s00259-023-06237-w

**Published:** 2023-04-26

**Authors:** Johanna S. Enke, Nic G. Reitsam, Tina Schaller, Rainer Claus, Constantin Lapa, Alexander Dierks

**Affiliations:** 1 Nuclear Medicine, Faculty of Medicine, University of Augsburg, Augsburg, Germany; 2 Pathology, Faculty of Medicine, University of Augsburg, Augsburg, Germany; 3 Internal Medicine, Faculty of Medicine, University of Augsburg, Augsburg, Germany

A 54-year-old man with newly diagnosed, histologically confirmed intraorbital marginal zone lymphoma (MZL) was referred for initial staging of disease. Whole-body ^18^F-fluordesoxyglucose ([^18^F]FDG) PET/CT was performed and showed only moderate uptake of the primary manifestation (A, red arrow) and mediastinal lymph nodes (A).

As [^18^F]FDG has limited sensitivity in the staging of MZL [1], an additional C-X-C motif chemokine receptor 4 (CXCR4)-directed PET/CT scan using [^68^Ga]Ga-PentixaFor ([^68^Ga]Ga-CPCR4.2) was performed, given that CXCR4 is overexpressed by most B- and T-cell neoplasms [2, 3]. Chemokine receptor-directed imaging demonstrated high tracer uptake of the intraorbital MZL-manifestation (B, red arrow) as well as multiple lymph nodes of the neck and thorax, the latter being rated as possible nodal MZL manifestations (B). Transbronchial fine-needle aspiration of a paratracheal lymph node (red star; [^18^F]FDG, SUV_max_ = 4.77; [^68^Ga]Ga-CPCR4.2, SUV_max_ = 7.18) revealed no signs of lymphoma infiltration but characteristic epithelioid cell granulomas with pronounced CXCR4-expression in the surrounding rim of activated lymphocytes (C), consistent with the diagnosis of sarcoidosis, a multisystem inflammatory disorder of enormous heterogeneity in clinical presentation [4].

Since CXCR4 is abundantly involved in immune cell activation and several inflammatory processes, and especially expressed by macrophages and T-lymphocytes [5], intense CXCR4-expression in sarcoid lesions is biologically reasonable. To our knowledge, this is one of the first reports on CXCR4 visualization in sarcoidosis by means of PET/CT. While sarcoidosis (as other inflammatory conditions) might represent a pitfall in oncologic imaging using CXCR4-directed PET tracers, non-invasive detection of receptor expression could also benefit the diagnostic workup of sarcoidosis, especially in cardiac- or neurosarcoidosis, and should be further evaluated.
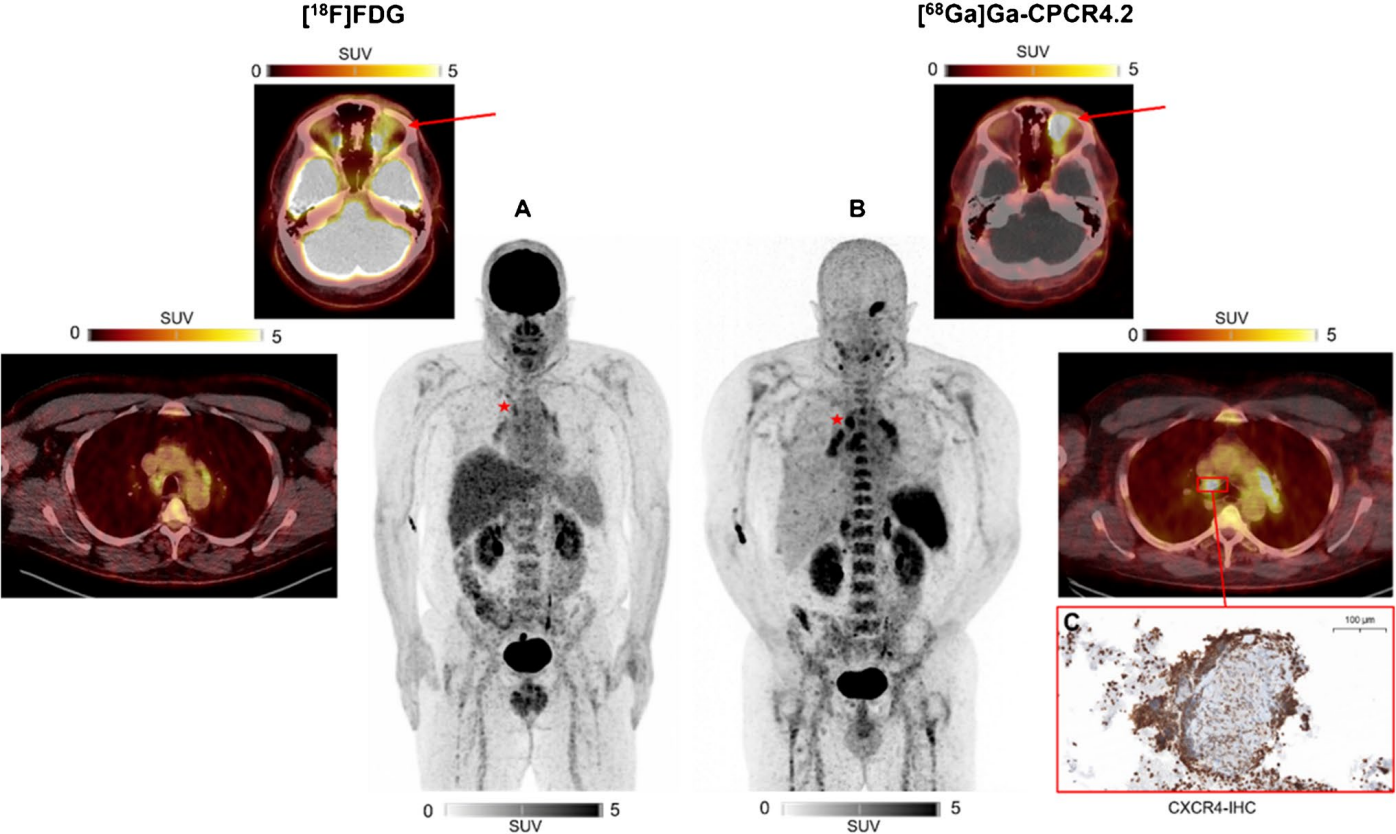


## Data Availability

The data that support the findings of this study are available from the corresponding author, [CL], upon reasonable request.
